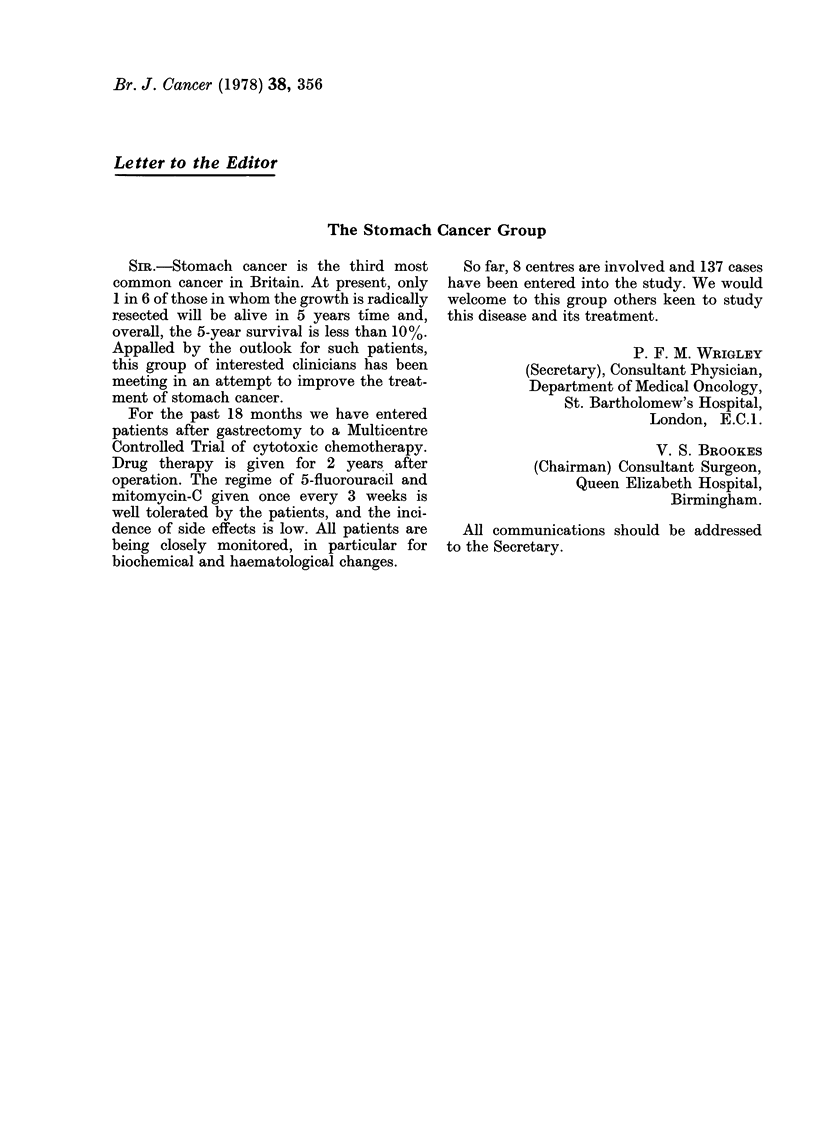# The Stomach Cancer Group

**Published:** 1978-08

**Authors:** P. F. M. Wrigley, V. S. Brookes


*Br. J. Cancer* (1978) **38**, 356

### *Letter to the Editor*

### The Stomach Cancer Group

SIR.—Stomach cancer is the third most common cancer in Britain. At present, only 1 in 6 of those in whom the growth is radically resected will be alive in 5 years time and, overall, the 5-year survival is less than 10%. Appalled by the outlook for such patients, this group of interested clinicians has been meeting in an attempt to improve the treatment of stomach cancer.

For the past 18 months we have entered patients after gastrectomy to a Multicentre Controlled Trial of cytotoxic chemotherapy. Drug therapy is given for 2 years after operation. The regime of 5-fluorouracil and mitomycin-C given once every 3 weeks is well tolerated by the patients, and the incidence of side effects is low. All patients are being closely monitored, in particular for biochemical and haematological changes.

So far, 8 centres are involved and 137 cases have been entered into the study. We would welcome to this group others keen to study this disease and its treatment.

P. F. M. WRIGLEY
(Secretary), Consultant Physician,
Department of Medical Oncology,
St. Bartholomew's Hospital,
London, E.C.1.

V. S. BROOKES
(Chairman) Consultant Surgeon,
Queen Elizabeth Hospital,
Birmingham.

All communications should be addressed to the Secretary.